# Evaluation of the Efficacy of Low-Particle-Size Toothpastes against Extrinsic Pigmentations: A Randomized Controlled Clinical Trial

**DOI:** 10.3390/dj11030082

**Published:** 2023-03-16

**Authors:** Andrea Butera, Maurizio Pascadopoli, Simone Gallo, Alessia Pardo, Giulia Stablum, Marco Lelli, Anna Pandolfi, Andrea Scribante

**Affiliations:** 1 Unit of Dental Hygiene, Section of Dentistry, Department of Clinical, Surgical, Diagnostic and Pediatric Sciences, University of Pavia, 27100 Pavia, Italy; 2 Unit of Orthodontics and Pediatric Dentistry, Section of Dentistry, Department of Clinical, Surgical, Diagnostic and Pediatric Sciences, University of Pavia, 27100 Pavia, Italy; 3 Dentistry and Maxillofacial Surgery Unit, Department of Surgery, Dentistry, Paediatrics and Gynaecology (DIPSCOMI), University of Verona, Piazzale L.A, Scuro 10, 37134 Verona, Italy; 4 Department of Industrial Chemistry «Toso Montanari», University of Bologna, 40126 Bologna, Italy

**Keywords:** oral hygiene, pigments, dentifrices, activated charcoal

## Abstract

Stain-removing domiciliary protocols are focused on the elimination of dental extrinsic pigmentations by the application of abrasive toothpastes, extensively available in commerce. The goal of the present study is to evaluate the efficacy of two different stain removal molecule-formulated toothpastes by the reduction of clinical parameters: the micro-cleaning crystals and activated charcoal. A total of 40 participants with extrinsic dental pigmentations were enrolled and divided into two groups: a Control group, assigned to a toothpaste with micro-cleaning crystals (Colgate Sensation White); and a Trial group, with microparticle-activated charcoal toothpaste (Coswell Blanx Black). At T0 (baseline), T1 (10 days), T2 (1 month), and T3 (3 months), clinical parameters, including Lobene stain index calculated for intensity and extension, plaque control record, and bleeding on probing, were measured. Statistically significant differences were found in both groups (*p* < 0.05): a reduction of extrinsic pigmentation, both in intensity and extension, was obtained in the Control group, but their total elimination could be achieved only in the Trial group with the activated charcoal molecule, though without significant difference between the groups (*p* > 0.05). No intergroup differences were found for each timeframe for PCR, BoP, LSI-I, and LSI-E. Both tested toothpastes can be recommended for domiciliary oral hygiene of patients with extrinsic pigmentations.

## 1. Introduction

Tooth discoloration is one of the most commonly reported complaints in patients seeking aesthetic treatment. Variations in the tooth colour can be influenced by intrinsic and extrinsic factors, ranging from chemical ingestion to consumption of foods that cause stains [1,2].

Extrinsic pigmentations lead to a change in natural tooth colour, and they are prevalently caused by the consumption of foods and drinks, such as tea, infusions, coffee, and tobacco use [3,4,5]. These stains appear on the enamel surface with various colours, ranging from light yellow/brown to very intense dark brown or black [6,7,8,9]. Professional tooth whitening with hydrogen peroxide and carbamide peroxide gel is considered an essential component of the intrinsic tooth stain eliminations, such as polishing for external tooth stain treatment [10]. 

Extrinsic pigmentations are related to the bacterial biofilm [11] and can be removed during professional oral hygiene sessions with mechanical debridement, polishing (using pastes with variable relative dentin abrasivity index—RDA) and air-polishing system techniques (using powders of different abrasiveness, such as glycine, bicarbonate, or calcium carbonate), in order to disrupt the bacterial biofilm and remove the stains [12]. 

Dental stains can also be removed at home using specific whitening toothpaste, with different abrasiveness and different stain-removing molecules [13,14].

In vitro and in vivo studies evaluating the effects of toothpaste have been reported in the literature, using different ingredients, such as surfactants, peroxide, enzymes, citrate, pyrophosphates and hexametaphosphate [10,12]. The primary stain removal ingredient tested in whitening toothpaste is the abrasive molecule. 

Studies have been conducted evaluating the effectiveness of activated charcoal against extrinsic pigmentations in vitro and in vivo [15,16]. The charcoal content in charcoal toothpastes is a fine powder form of activated charcoal that has been oxidized by controlled heating or by chemical treatment. It is obtained through a method called “slow pyrolysis”, removing water and other volatile components from carbon-rich materials. Substances such as nut shells, coconut husks, or peat, are heated in an oxygen-free environment [10,17]. The present study aims to test the stain-removing action of activated charcoal, compared to micro-cleaning crystals.

Therefore, the primary aim of the study is to evaluate the effectiveness against extrinsic pigmentations of a toothpaste containing micro-particles of activated charcoal, compared to a toothpaste containing micro-cleaning crystals, which is a technology commonly used for domiciliary dental hygiene products [18]. 

The secondary aim of the study is to evaluate the efficacy of the tested toothpastes in the improvement of plaque and bleeding indices over time. Accordingly, the first null hypothesis is that no difference occurs between the two tested toothpastes for the stain removal efficacy, evaluated through the Lobene stain index extension and intensity (LSI-E and LSI-I). The second null hypothesis is that the two products cause no significant alterations in the plaque control record (PCR). 

The third null hypothesis is that no significant differences occurred between bleeding on probing outcomes. 

## 2. Materials and Methods

### 2.1. Study Design

This was a double-arm parallel, active-controlled, randomized clinical trial (RCT), with a 1:1 allocation ratio. The study was conducted in accordance with the Declaration of Helsinki on experimentations involving human subjects. The current trial was conducted based on the CONSORT statement and approved by the Unit Internal Review Board (registration number: 2021-0519). The protocol was registered on clinicaltrials.gov (NCT: NCT04904978). Written informed consent was obtained from all the participants involved. The study started in May 2021 and ended in April 2022. 

### 2.2. Participants

Patients addressing for routinary periodontal care at the Unit of Dental Hygiene, Section of Dentistry, Department of Clinical, Surgical, Diagnostic and Pediatric Sciences, University of Pavia, 27100 Pavia, Italy, were enrolled. The inclusion criteria adopted for the study were:

- Adult patients
- Presence of extrinsic stains

The following exclusion criteria, detected on the basis of the preliminary clinical assessment, were considered:

- Underage patients
- Patients suffering from neurological or psychiatric disorders
- Pregnant women
- Patients undergoing anticancer chemotherapy

### 2.3. Interventions and Outcomes

At the baseline (T0), patients were probed with a periodontal probe (UNC probe 15; Hu-Friedy, Chicago, IL, USA) and the subsequent indexes were evaluated on each tooth:

- PCR—plaque control record by O’Leary [19] allows to assess the compliance of the patient and their level of oral hygiene at home by identifying the areas of biofilm accumulation. Each tooth was divided into four surfaces: mesial, distal, vestibular, palatal/lingual. The presence/absence of plaques for all surfaces was recorded. PCR is therefore calculated as a percentage value, dividing the number of plaque-containing surfaces by the total number of available surfaces, multiplied by 100.
- BoP—bleeding on probing [20] allows to assess the level of gingival inflammation, calculated after gentle probing with a periodontal probe. All teeth underwent periodontal probing on the three buccal surfaces (mesiobuccal, medial and distobuccal). BoP was considered positive when bleeding occurred 20 s after probing. As for the PCR, a percentage value was obtained dividing the total positive sites by the total sites, multiplied by 100.
- LSI—Lobene stain index [21] allows to evaluate the intensity (LSI–I) and extension (LSI–E) of the extrinsic pigmentations on the vestibular or palatal/lingual surface of teeth presenting extrinsic stains. The allocation grade method is summarised in Table 1.

Then, patients were randomly divided by the same operator (not involved in further clinical or research procedures) into two different groups:

- Control group: patients had to use Colgate^®^ Sensation White (Colgate-Palmolive, New York, NY, USA) for home oral care twice a day
- Trial group: patients had to use Blanx Black^®^ (Coswell S.p.A., Funo di Argelato, BO, Italy) for home oral care twice a day

The compositions of the two toothpastes are shown in Table 2.

Patients were evaluated after 10 days (T1), one month (T2) and 3 months (T3) to evaluate the efficacy of the assigned treatment. The complete clinical protocol is described in Table 3.

Adverse effects were evaluated if enamel discoloration and mucosal ulceration and/or inflammation were present.

Operator calibration was obtained by performing the evaluation of the same quantitative parameters at a 14-day distance by the same operator. 

### 2.4. Sample Size

Sample size calculation was performed assuming type I error alpha = 0.05 and type II error power = 80% for two independent study groups, and a continuous primary endpoint. The primary outcome chosen was “Lobene index intensity”. The following mathematical formula was used for the two-tailed sample size calculation:

$$\mathrm{Sample}\;\mathrm{size}=\frac{Z_{\left(1-\frac{\alpha }{2}\right)}^{2}p\left(1-p\right)}{d^{2}},$$

where $z_{\left(1-\frac{\alpha }{2}\right)}$ is the standard normal variate corresponding to 1.96 at 5% type 1 error, *p* is the expected proportion in population expressed as a decimal and based on previous studies, and finally *d* is the confidence level decided by the researcher and expressed as a decimal. Based on results of Yin et al. [22], an expected value of 1.10 was hypothesized. The expected difference between the means was supposed to be 0.41, with a standard deviation of 0.46; therefore, 20 patients per group were required for the study. 

### 2.5. Randomization and Blinding 

A block randomization table was used in order to allocate patients, and a randomization sequence was provided by the statistician, considering a permuted block of 40 total participants. Another operator, not previously involved in the procedures, enrolled the participants and executed the professional oral procedures, and collected the outcomes. On the basis of previously prepared sequentially numbered, opaque, sealed envelopes (SNOSE), an assistant assigned each participant to the respective group, concealing the products for home use. The data analyst was blinded for the allocation and outcomes. For the domiciliary oral hygiene procedures, the two products tested were concealed. Neither the operator nor the patients were aware of the treatment administered. 

### 2.6. Statistical Analysis

Data were submitted to statistical analysis with the R software (R version 3.1.3, R Development Core Team, R Foundation for Statistical Computing, Wien, Austria). For each group and variable, descriptive statistics including the mean, standard deviation, median, minimum, maximum, and interquartile range (for continuous variables) were measured for each group. Data normality was assessed with the Kolmogorov–Smirnov test (PCR: *p* < 0.0285; LBI-E: *p* < 0.0013; LBI-I: *p* < 0.0012; BoP: *p* < 0.0423). As data were not normally distributed for all the variables tested, a Friedman’s test for repeated measures was performed, followed by Dunn’s post hoc test. The K statistic was calculated to assess intraclass correlation coefficient (ICC) by performing the same parameter evaluation after 2 weeks by the same operator. Statistical significance was predetermined for *p* < 0.05 for all statistical tests.

## 3. Results

### 3.1. Participant Flow and Baseline Data

**Figure 1:**
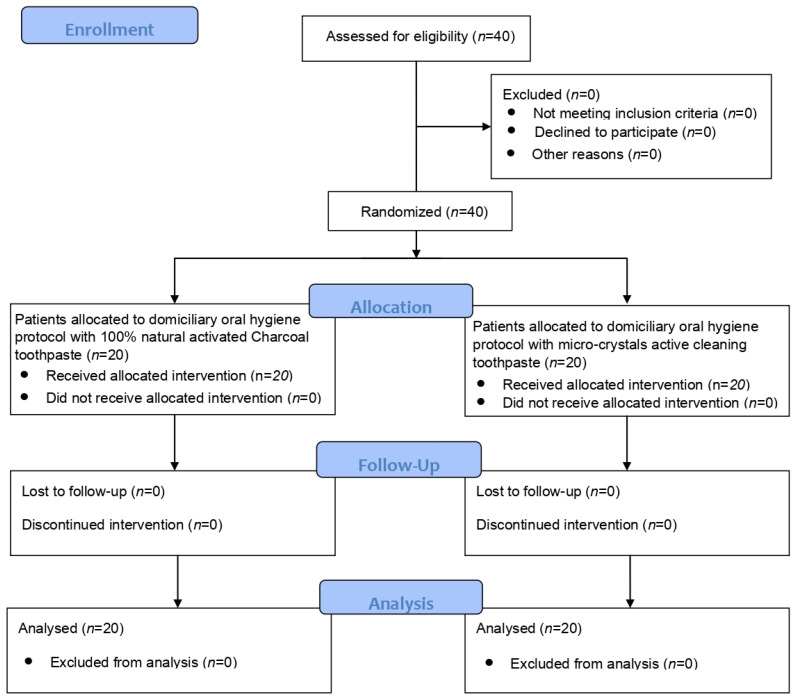
CONSORT flow chart.

Figure 1 shows the CONSORT flow chart of the study. After screening, 40 patients fulfilling the inclusion criteria were recruited. At the end of the last follow-up (T3), all patients completed the study. 

At baseline, the demographic data of the study sample was a mean age of 34.7 ± 10.6 years (17 males and 23 females; 20 patients for the Trial group, mean age 31.7 ± 10.04; 20 patients for the Control group, mean age 37.7 ± 10.51). 

The descriptive and inferential statistics for each variable are represented below. A letter-based representation was adopted to highlight significant inter- and intragroup differences.

K statistics revealed an intraclass correlation coefficient (ICC) of 0.83.

### 3.2. Plaque Control Record

PCR scores are presented in Table 4. The scores decreased from T0 and T3 in both groups. Statistically significant intragroup differences were found comparing T0–T2 and T0–T3 time frames in the Control group (*p* < 0.05). Conversely, in the Trial group, significant differences were found between T0 and T2, T0 and T3, and T1 and T3 (*p* < 0.05). No intergroup differences were assessed for each timepoint (*p* > 0.05). 

### 3.3. Bleeding on Probing

BoP scores are shown in Table 5. The scores decreased from T0 and T3 in both groups. Statistically significant intragroup differences were found comparing T0–T2 in the Control group (*p* < 0.05), and T0–T3 and T1–T3 for the Trial group (*p* < 0.05). No significant intergroup differences were assessed for each time frame (*p* > 0.05). 

### 3.4. Lobene Stain Index-Intensity

LSI-I scores are shown in Table 6. The scores decreased from T0 and T3 in both groups. Statistically significant intragroup differences were found comparing T0–T3 and T1–T3 in the Control group (*p* < 0.05), and T0–T2, T0–T3, T1–T3 and T2–T3 for the Trial group (*p* < 0.05). No significant intergroup differences were assessed for each time frame (*p* > 0.05).

### 3.5. Lobene Stain Index-Extension

LSI-E scores are shown in Table 7. The scores decreased from T0 and T3 in both groups. Statistically significant intragroup differences were found comparing T0–T3 and T1–T3 in the Control group (*p* < 0.05), and T0–T2, T0–T3, T1–T3 and T2–T3 for the Trial group (*p* < 0.05). No significant intergroup differences were assessed for each time frame (*p* > 0.05). 

### 3.6. Harms and Adverse Effects

No harms or adverse effects were reported during the study.

## 4. Discussion

Although professional in-office treatments, including polishing and air-polishing system, represent the gold standard for the extrinsic dental pigmentations therapy [3], the domiciliary use of whitening toothpastes can successfully aid their removal at home. Currently, there are several products on the market that remove stains and claim to whiten teeth [1]. 

Some toothpastes additionally contain anti-plaque and anti-gingivitis ingredients, such as lactoferrin, making these products (together with the fluoride) multi-functional [23,24]. The development of these products is not straightforward because of the interaction between formulation components; moreover, the active ingredients should maintain their beneficial characteristics during the shelf-life of the paste [25]. Until now, different studies evaluated the domiciliary effect of activated charcoal on extrinsic pigmentations [10,15]. 

Previous studies have been conducted in the literature to evaluate the effects on stains by both crystal- [26,27] and charcoal-based toothpastes [28,29], highlighting positive results for the two methodologies. The current research was aimed at comparing both agents at the same time. 

The first null hypothesis of the study has been accepted, as no significant differences occur between the two groups per each time frame. Concerning the LSI-E and LSI-I, it can be stated that the Control group showed a significant reduction in pigmentation for both the indexes. After three months (T3), a higher stains removal was achieved in the Trial group, with no significant difference. 

The secondary objective of the study was to evaluate PCR and BoP indices for the two toothpastes. The second and the third null hypotheses of the study have been partially rejected, as no significant differences were found between the two groups. In detail, the PCR index underwent a reduction of more than 50% in the trial group, probably due to the charcoal molecules, which, with their structure composed of 80% cavities, are hypothesized to capture plaque and other impurities that are deposited within them [30]. Significant results also emerged for the BoP and in both groups, a progressive decrease can be evidenced: in the Control group, the comparison results of T0–T3 were not significant, while the same did not occur in the Trial group. Even though the Trial group exhibited lower values of BoP and PCR compared to the Control group at T3, with lower mean values of BoP and PCR in the Trial group, no statistically significant difference was found between them. 

The research on the anti-stain toothpastes has been particularly extensive in recent years, with a high number of new molecules tested, such as sodium polyaspartate, silica, sodium phytate, and sodium pyrophosphate [31,32]. Different results were obtained, depending on the specific component, but direct comparisons are difficult to make due to the heterogeneous methodologies in the studies.

Regarding the studies conducted on the efficacy of crystals on stain removal, Putt and colleagues [33] evaluated the efficacy of the removal of existing extrinsic tooth stains of a sodium bicarbonate (baking soda), dual-phase dentifrice containing calcium and phosphate (Trial Dentifrice), with respect to a commercially hydrated silica dentifrice (Control Dentifrice), during a 6-week normal use. The trial toothpaste demonstrated statistically significant efficacy in removing naturally acquired, extrinsic tooth stain, and appeared to be significantly more effective for stain removal than a commercially available hydrated silica dentifrice. 

Considering the efficacy of charcoal as a whitening agent, Dursun and colleagues [34] evaluated the action of abrasives, polyphosphates, activated charcoal, and hydrogen peroxide, demonstrating an analogue effect among them. Considering that the study mentioned above was conducted in vitro, no direct comparison could be conducted with the present report. Vertuan and colleagues [35] concluded that toothpastes containing charcoal, combined with pyrophosphate, might have a high abrasive effect on eroded tooth surfaces, therefore care should be taken by patients when using these products, as they may show a high abrasive effect. However, in another in vitro study, the charcoal-containing dentifrices were shown to be abrasive within acceptable limits set by the ISO [36]. In a recent systematic review of in vitro studies, it was concluded that toothpastes based on activated charcoal have a lower whitening effect than other alternatives, and can be considered as less safe due to its high abrasive potential [37]. However, the results of this study cannot be directly referred to an in vivo condition. The lack of clinical trials on this this topic justifies the purpose of the current clinical trial. 

Based on the results obtained in the present study from the two groups which were comparable, it can be stated that both the molecules tested are effective in reducing extrinsic pigmentations at home, as well as in reducing plaque and bleeding indexes, with no significant intergroup differences between the two. Based on this consideration, the present study showed that both the use of a toothpaste containing activated charcoal and a toothpaste containing conventional micro-cleaning particles are effective as oral hygiene products and as external bleaching agents. It would be interesting to evaluate the use of the two molecules mentioned above with other products in further studies, in order to detect an eventual synergic action. 

In addition to the ones mentioned above, a relevant limitation of the study is that patient treatment of the domiciliary could have been biased. Moreover, a 3-month follow-up could be relatively short in the perspective of a long-term whitening. Finally, the calibration process of the operators was performed for quantitative variables; however, even qualitative variables have been considered in this study and, as an intrinsic limit, could not undergo a calibration process. 

Future randomized clinical trials with a longer follow up, and trying to standardize the domiciliary intervention assigned as much as possible, are needed. The comparison of the toothpastes tested in this study with others available on the market based on different chemical compounds (e.g., sodium polyaspartate, silica, sodium phytate, and sodium pyrophosphate) would also be interesting, in order to find the most appropriate domiciliary protocol for both the oral hygiene and tooth whitening. Finally, as future perspectives, it would be interesting to combine charcoal with probiotic-based products, such as para-probiotics (heat-inactivated bacteria) [38], lysates (bacterial fragments) [39] and postbiotics (concentrated bacterial active metabolites) [40], which all showed promising results in clinical dentistry. Accordingly, future research is needed in order to improve current knowledge about these treatment possibilities in combination with charcoal.

## 5. Conclusions

The present study showed that both the use of a toothpaste containing activated charcoal and a toothpaste containing conventional micro-cleaning particles significantly reduce extrinsic pigmentations when used for a domiciliary protocol. Successful results were also obtained for the reduction of plaque and bleeding indexes, with no significant intergroup differences.

## Figures and Tables

**Table 1:** Modified Lobene Index.

Grade	Intensity	Extension
0	Absence of pigmentations	Absence of pigmentations
1	Light spot ranging from yellow to light brown–grey	Pigmentations on more than one third of the area
2	Moderate brown spot	Persistence of the pigmentations from one third and two thirds of the assessed area
3	Dark spot ranging from brown to dark brown	Pigmentations on more than two thirds of the area evaluated

**Table 2:** Compositions of the tested toothpastes.

Gel	Manufacturer	Composition
Blanx Black^®^	Coswell S.p.A., Funo di Argelato, BO, Italy	Limonene, aqua, sodium lauryl sulfate, glycerin, sorbitol, silica, cellulose gum, charcoal powder, sodium monofluorophosphate, hydrated silica, aroma, xylitol, cetraria islandica extract, papain, maltodextrin, benzyl alcohol, sodium saccharin, usnea barbata extract, sodium benzoate, phenoxyethanol.
Colgate Sensation White^®^	Colgate-Palmolive, New York, NY, USA	Aqua, aroma, hydrated silica, sodium fluoride, sorbitol, sodium saccharin, polyethyleneglycol-12, sodium bicarbonate, sodium lauryl sulfate, cellulose gum, limonene, CI 77891, xanthan gum, CI 74160.

**Table 3:** Protocol considered for the study.

Appointment	Procedures
	Signature of the informed consent for the study
	Assessment of periodontal clinical indexes
	Professional supragingival and subgingival oral hygiene
Baseline (T0)	Supragingival and subgingival decontamination with glycine powders Motivation for oral hygiene and instruction for the domiciliary treatment: *Trial Group*: Colgate^®^ Sensation White toothpaste (Colgate-Palmolive, New York, NY, USA) *Control Group*: Blanx^®^ Black toothpaste (Coswell S.p.A., Funo di Argelato, BO, Italy)
After 10 days (T1) After 1 month (T2) After 3 months (T3)	Assessment of clinical indexes Further motivation for oral hygiene and progression of the domiciliary treatment assigned

**Table 4:** Descriptive statistics of PCR, and significant intragroup and intergroup differences assessed by the Dunn’s post hoc test. * The means with the same letters are not significantly different (*p* > 0.05).

Group	Time	Mean	St. Dev.	Min.	Max.	Median	Interquartile Range	Significance *
Control (crystals)	T0	78.75	25.07	25.00	100.00	80.00	20.00	A
	T1	63.50	31.71	15.00	100.00	65.00	70.00	A, B, C
	T2	49.75	24.14	10.00	80.00	50.00	42.50	B, C, D
	T3	44.50	27.76	10.00	80.00	50.00	51.25	B, C, D
Trial (charcoal)	T0	72.75	29.67	20.00	100.00	80.00	46.25	A, B
	T1	43.25	20.98	10.00	80.00	50.00	36.25	B, C
	T2	31.75	17.49	10.00	80.00	25.00	21.25	C, D
	T3	17.75	10.19	5.00	50.00	20.00	10.00	D

**Table 5:** Descriptive statistics of BoP, and significant intragroup and intergroup differences assessed by the Dunn’s post hoc test. * The means with the same letters are not significantly different (*p* > 0.05).

Group	Time	Mean	St. Dev.	Min.	Max.	Median	Interquartile Range	Significance *
Control (crystals)	T0	13.50	9.33	0.00	30.00	10.00	10.00	A, C
	T1	8.50	7.96	0.00	20.00	5.00	16.25	A, B, D
	T2	5.00	5.13	0.00	15.00	5.00	10.00	B, D
	T3	6.75	3.73	0.00	10.00	7.50	5.00	A, D
Trial (charcoal)	T0	28.25	27.92	0.00	80.00	20.00	26.25	C
	T1	11.50	10.14	0.00	30.00	10.00	12.50	A, B, C
	T2	6.75	6.34	0.00	20.00	7.50	10.00	A, B, D
	T3	3.25	4.38	0.00	10.00	0.00	6.25	D

**Table 6:** Descriptive statistics of LSI-I, and significant intragroup and intergroup differences assessed by the Dunn’s post hoc test. * The means with the same letters are not significantly different (*p* > 0.05).

Group	Time	Mean	St. Dev.	Min.	Median	Max.	Significance *
Control (crystals)	T0	1.85	0.67	1.00	2.00	3.00	A, B
	T1	1.70	0.66	1.00	2.00	3.00	A, B
	T2	1.15	0.75	0.00	1.00	2.00	B, C, D, E
	T3	0.85	0.49	0.00	1.00	2.00	D, E
Trial (charcoal)	T0	2.25	0.64	1.00	2.00	3.00	A
	T1	1.85	0.81	0.00	2.00	3.00	A, B, C
	T2	1.30	0.66	0.00	1.00	3.00	B, C, D
	T3	0.35	0.49	0.00	0.00	1.00	E

**Table 7:** Descriptive statistics of LSI-E, and significant intragroup and intergroup differences assessed by the Dunn’s post hoc test. * The means with the same letters are not significantly different (*p* > 0.05).

Group	Time	Mean	St. Dev.	Min.	Median	Max.	Significance *
Control (crystals)	T0	1.90	0.72	1.00	2.00	3.00	A, B
	T1	1.60	0.68	1.00	1.50	3.00	A, B
	T2	1.15	0.49	0.00	1.00	2.00	B, C, D
	T3	0.80	0.52	0.00	1.00	2.00	D, E
Trial (charcoal)	T0	2.05	0.60	1.00	2.00	3.00	A
	T1	1.60	0.68	1.00	1.50	3.00	A, B, C
	T2	1.25	0.44	1.00	1.00	2.00	B, C, D
	T3	0.25	0.44	0.00	0.00	1.00	E

## Data Availability

Data are available upon reasonable request to the corresponding authors.
